# Corrigendum to “Cost-effectiveness of intraoperative transesophageal echocardiography in cardiac valve surgery” [J Cardiovasc Thorac Res 2011;3(3):79-81]

**DOI:** 10.5681/jcvtr.2014.016

**Published:** 2014-03-21

**Authors:** Bahman Naghipour, Rasoul Azarfarin, Samad EJ Golzari, Moussa Mirinazhad, Eissa Bilehjani, Sohrab Negargar

**Affiliations:** ^1^Department of Anesthesiology, Tabriz University of Medical Sciences, Tabriz, Iran; ^2^Cardiovascular Research Center, Tabriz University of Medical sciences, Tabriz, Iran; ^3^Medical Philosophy and History Research Center, Tabriz University of Medical Sciences, Tabriz, Iran

The authors would like to correct the name of the third author in this paper to Samad EJ Golzari.

